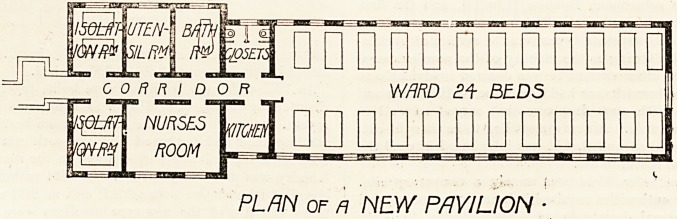# Some Modern Continental Hospitals

**Published:** 1908-11-07

**Authors:** 


### HOSPITAL ADMINISTRATION.

### CONSTRUCTION AND ECONOMICS.

### SOME MODERN CONTINENTAL HOSPITALS.

#### I.—THE MOABITE HOSPITAL, BERLIN.

THE EPIDEMICS OF BERLIN.

BERLIN, as a city, has suffered much from epidemic visitations. Cholera, scarlet fever, small-pox, and even rheumatic fever have periodically frightened the city fathers, especially in the days before the Austrian and Franco-Prussian wars. The hospital accommodation was insufficient for the needs of the various quarters, and during an epidemic it was absolutely inadequate, so that the municipality was forced to establish temporary hospitals with all their attendant evils. Thus during the cholera epidemic of 1866, three private houses were bought and turned into temporary hospitals, in which no fewer than 2,600 patients were treated within three months. The mortality and the great difficulties attending the working of these temporary infirmaries made the authorities seriously consider the question of providing suitable quarters for the sick in case of another epidemic. Nor did they have long to wait for the necessity for immediate action to arise. In 1871—a strenuous year in the annals of Berlin, as in those of Germany as a whole—came the small-pox epidemic, and the urgent need for hospitals was keenly felt. Most towns tried to meet this need by constructing temporary lazarets on the principle of those in use in the army and abroad. Thus a frequent type to copy was the Hampstead small-pox lazaret—later turned into the North-Western Hospital, which in its turn has become the Hampstead General Hospital—which consisted of large pavilions, each containing thirty-four beds; or the St. Pauli Hospital at Hamburg, whose pavilions were cheaper, smaller, and more like those used in the army. Modifications of these two primary types readily suggested themselves, and a number of such modified barrack pavilions was finally erected on the Tempelhofer Field at Berlin.

THE TEMPELHOFER TYPE.

The Tempelhofer type was destined to become the type for the subsequent Moabite Hospital, and the first point that emphasised the difference between it and the St. Pauli and Hampstead types was the system of ventilation adopted. Owing to the vicinity of the powder magazines and for other reasons it was deemed inadvisable to provide these barracks, as had been done at Hamburg and Hampstead, with open fireplaces that served so well to ventilate the wards. A new system had to be thought out, and it answered so well that it was introduced into all the pavilions, and is still in use.

The Tempelhofer Field was merely a temporary site. The military authorities required it for army purposes, and as it had several drawbacks as a hospital site, the City Council was forced to consider a new scheme. In December 1871 a special committee was appointed to consider fully the whole question of temporary hospital accommodation in times of epidemic, and to report on the advisability of erecting a series of new barracks of the Templehofer type elsewhere. This committee, which consisted of Prof. Virchow, Architect Gerstenberg, and Councillor Meyer, recommended that the Council should proceed at once with the erection of a temporary hospital, of the barrack type, on a strip of vacant ground, communal property, and 759,000 square metres in extent, lying close to the Turmstrasse, in the Moabite quarter, at that time practically on the outskirts of Berlin. On this strip of ground three barracks of the Tempelhofer type had already been erected early in the year, and the Committee advised that 16 new pavilions should be built at once into which the patients located in the Tempelhofer Field barracks could be forthwith removed. The report was adopted by the Council, 150,000 marks was voted (a further sum of 450,000 marks was granted in 1872), and the work was started on January 2, 1872.

RAPID HOSPITAL CONSTRUCTION.

The workmen laboured day and night, and by the end of March, 1872, the hospital, as designed, was finished and ready for the accommodation of patients. Quicker, and at the same time more thorough, work it would be difficult to find in the history of hospital construction. Not only the sixteen barracks, but an administrative block, a laundry, kitchen, mortuary, and adjacent buildings were completed within the short space of less than three months, and the proof of the excellence of the workmanship is to be found in the fact that of the pavilions then built several are still standing and being used, those that are no longer extant having been deliberately pulled down to make room for the newer wards. As completed then the hospital cost the astonishingly low sum of about £30 per bed.

In 1873 eight new pavilions were built, together with a disinfecting house. In 1875 an ice-house was added and several outhouses; 1879 the central steam-heating system was modified and improved and the park was laid out; 1883 came the isolation barracks and in the same year the laundry was practically rebuilt. In 1884 pathological and chemical laboratories were added, and in 1889 a new mortuary and five of the new-type pavilions were built. In 1890 the hospital had altogether thirty pavilions with 828 beds. In 1896 the present operating theatre was constructed, Pavilion 12 having hitherto served as such. In 1895-99 extensive alterations and additions were made.

The ground plan of the old hospital shows very clearly the arrangement of the pavilions. The hospital block was in the shape of a rectangle with its diagonal running east and west with the angles rounded off, and a long oval park in the centre, round which, in the form of a semi-oval with converging horns, were arranged the thirty pavilions. The main entrance was then, as it is now, in Thurmstrasse, and on this side lay the administrative block, the dwelling houses, the laundry and kitchen. On the right-hand side of the rectangle, behind the right-hand pavilions, lay the mortuary, chapel, post-mortem room, pathological and chemical laboratories and kennels. The park was tastefully laid out with shrubs and trees, and the pavilions, in summer time at least, were embowered among foliage and greenery. Between each pavilion was a wide street which led into the still wider semi-circular thoroughfare which ran in front of the line of pavilions round the entire park. Partly the hospital was surrounded by a wall and in part by an iron-railing fence. At that time, of course, it lay to a large extent extra-urban, but Berlin has spread itself so much that the Moabite is now in one of the liveliest quarters of the city and as much an urban hospital as St. Thomas' or St. Bartholomew's.

MOABITE HOSPITAL·BERLIN·

PLAN OF AN OLD PAVILION·

PLAN OF A NEW PAVILION·

THE MOABITE HOSPITAL OF TO-DAY.

The present hospital does not differ, in its ground plan, very greatly from the old one, and the main additions are the five new pavilions erected behind the old ones at the extreme right, close to the nurses' home. In perspective view there would of course be many differences, seeing that the old administrative and economic blocks have been entirely rebuilt. The hospital is in the form of a rectangle, the smaller sides facing due north and south, so that the individual pavilions, at right angles to the longer sides, look east and west, a position which is open to criticism on several grounds. The total area is nearly 91,000 square metres, of which more than 15,000 square metres are occupied by the fifty-three buildings. On the Turmstrasse side lie the administrative block, the nursing home, kitchen and laundry blocks, all quite new—imposing red-brick buildings with cement facings and ornamental mouldings. The style of architecture is similar to that of the administrative block at the Virchow—a modification of French renaissance—and the responsible architect is Herr Hoffmann, who designed the latter hospital. Facing the administrative and economic blocks is the park with its long street of thirty pavilions arranged in horseshoe shape, with the five new pavilions lying behind the others on the left.

The old pavilions, some of which date back to 1871, are low, one-storied barracks, each one separated by a street and lawn stretch 17.5 metres wide from its neighbour, and having the whole stretch of the park and oval street—78 metres—between it and the opposite row. The five new pavilions lie 10 metres behind the old ones. The roof is of thin deal, double, asphalted outside, and sloping down for a considerable distance. The end doorway faces the "pavilion street," and through it one enters the ward at once. This is a large, airy room, measuring 28.25 metres by 6.90 metres by 4.7 metres, with twenty-eight windows—fourteen on each side—and thirty beds. The roof, sloping upwards as in a loft—there being no ceiling—is formed by the lower layer of deal, painted a rather sombre grey, with wooden girders running across from wall to wall, and special ventilators along the centre. The floor is, in most pavilions, of terrazzo, usually on a single foundation, but in some of the wards it is of tiles, blue and white alternating in a pleasing pattern. The terrazzo floor has proved very successful on the whole, being much less liable to cracks with a single foundation than where it has been laid down with a double layer of cement underneath as in most hospitals. It is somewhat easier to clean than the tiled floor, the interstices between the tiles needing wet sand and scrubbing for their successful cleaning. The ward is heated throughout by low pressure steam pipes which run along the sides, and the ventilation is insured by fourteen specially designed open ventilators in the centre of the roof. The warm, pure air enters at the bottom, along the sides, and passes upwards, escaping through these ventilators. The system, which is splendidly simple, is as splendidly efficient, and it is claimed that it insures an equable temperature and an uninterrupted pure atmosphere in the ward. On the first occasion when we visited the wards the outside temperature was some degrees above freezing point, wet, damp, and miserable, and it was a distinctly novel experience to enter a fireless ward, where the door stood wide open, and to find the atmosphere beautifully warm without any trace of stuffiness or foulness. On another occasion, when the morning was an unusually hot one, the ward was equally comfortable, all the windows being open. In summer the steam pipes are shut off and the ventilation is efficiently carried on through the windows.

### The Ward Arrangements.

The wards are extremely well arranged, and look cosy and homelike. This impression is largely due to the various little adornments that are found in it. From a hygienic point of view the neat pale yellow dimity curtains with their frilled top border round each window, the fern-baskets that hang from the roof over the two-metre wide corridor between the bed rows, and the pot plants arranged in clusters on stands here and there may perhaps be undesirable, but there is no doubt that they add very greatly to the cheerfulness of the wards and that they may have a psychological value which outweighs their hygienic disadvantages. The extreme cleanliness and care that prevails may be imagined when it is stated that the curtains were absolutely dustless and the pot plants and their stands as spick and span as if they had just been washed for a flower show. There are no curtains round the beds, as in many English hospitals, the necessary privacy when occasion requires being afforded by simple and light steel tube framed screens with striped calico blinds that can be interchanged for washing purposes. This screen, the Moabite model, is now in general use in most German hospitals, and is certainly an improvement upon our heavy, permanent types. The official calculation for each bed is 6.50 square metre surface area, 2.10 square metre light area (there being 63-metre window surface in each ward), and 25.5 cubic metre air space. This last may appear to be rather low, but is quite efficient when it is remembered that the ward is comparatively a "shallow" one and that the ventilation is exceptionally good. In hospitals where a larger calculation is given the roof is usually much higher and it is doubtful if the patients really benefit by the theoretical increase of cubic air space.

The beds themselves are worth noticing. Each bedstead is of light, tubular steel construction, 200 cm. long, 88 cm. wide, and 50 cm. high at the sides. On this is placed a special horsehair mattress, with spiral steel springs, a special "Moabite model" which is comfortable, elastic, and perfectly smooth. These mattresses, which are "needled" and covered with grey tick, cost 60 marks a-piece, but are very durable and can be easily sterilised by dry steam. Great attention is paid to the horsehair, which is specially examined by the hospital chemist before it is accepted. The hair must be undyed, averaging 25 cm. in length and able to bear an average weight of 400 gram. with a stretch of 7.5 per cent. of its net length. Over each mattress is stretched a Molton blanket; over this again a mackintosh sheet, followed by a double linen sheet and the usual woollen blanket. The bolster is of horsehair covered with tick; the pillow of goose feathers, which can be sterilised by dry heat. Alongside each bed is a small enamelled steel table; at the head the usual bed-plate. The cupboards in the ward are of glass and enamel ware, usually on wheels, and very little woodwork is to be seen.

### Time-saving Appliances.

The visitor will note with interest the many simple but effective appliances in the ward for lightening work and adding to the patients' comfort. These are the so called Moabite models—the special raising apparatus, a light steel contrivance with pulleys and a draw-sheet by means of which a helpless patient may be raised to allow the necessary changing of bed linen, dressings, etc., to be carried out with the least discomfort—the cheap yet strong invalid chairs, the special beds and mattresses for paralytic patients, the sliding children's cots, and the various other modifications which space forbids us describing in detail. One, however, demands a line, namely, the patient's transport wagon, a neat, light, rubber-tyred carriage on which the patient can be slipped and removed from the ward. An exactly similar carriage is used for the transport of bodies to the mortuary, a point which is worth attention, as it prevents the painful impression which the appearance of a bier or "corpse stretcher," such as is still used in some London hospitals, creates in a ward.

At the far end of the ward is the simple accessory block which constitutes the rear part of the pavilion. This consists of a central corridor leading into a broad but rather short day-room, a comfortable appartment with two large side windows overlooking the park. Then, proceeding farther down the corridor, one finds on the left-hand side the kitchen and the sisters' sitting-room, and on the left hand the lavatory and the large room for foul linen. The lavatory is simply arranged, but very effective, comprising a large bath-room with transportable baths and a shower-bath, a hand lavatory, and, quite separate from these, two water-closets. These closets are of the English type, the flushing stream being unusually large and powerful. Here, as in the ward, the ventilation is excellent, and everything scrupulously clean. The drains are large, and can be readily examined, and there is an entire absence of any foul or musty odour. The new pavilions have a double room on each side, just before one enters the ward. Here is the day room, which is comparatively small, but cosy and cheerful. Next to it is a scullery, disinfecting-room, utensil-room, and a small extra room. The disinfecting-room is fitted with special disinfecting apparatus, a steam disinfector, a soda-soap copper rinser, and a flushing boiler. A short corridor between these rooms leads directly into the ward, which contains twenty-four beds arranged between twenty-four windows. The calculated air, light, and floor space for each bed is therefore somewhat greater, the ward measuring the same as in the old pavilions. The general appearance of the ward is similar to that of the old pavilions, the floors being usually of terrazzo on a single foundation, and the walls painted. The corridor at the end gives access to the closets and lavatory on the right, and the kitchen on the left-hand side, as in the old pavilions. Then, on the left-hand side, come a large room for the nursing staff, and an isolation room with two beds. On the opposite side is a bath-room, a utensil-room, or really a mop-room, and a similar two-bedded isolation room.

Each new pavilion of this Moabite Hospital, therefore, has accommodation for twenty-eight instead of thirty patients. In construction the old and the new pavilions are similar, consisting of a simple brick building, lined inside with thin deal painted grey, and resting on a specially constructed cement foundation. The drainage has been carefully attended to, and is thoroughly efficient, being connected with the main city drainage. In the old pavilions gas-light is used; in the new ones electricity is replacing it. The average cost of the new barrack pavilions was 27,000 marks, or just below £50 per bed. Altogether the hospital accommodates close upon 1,000 patients, of whom the greater part—more than 700—are medical.

## Figures and Tables

**Figure f1:**
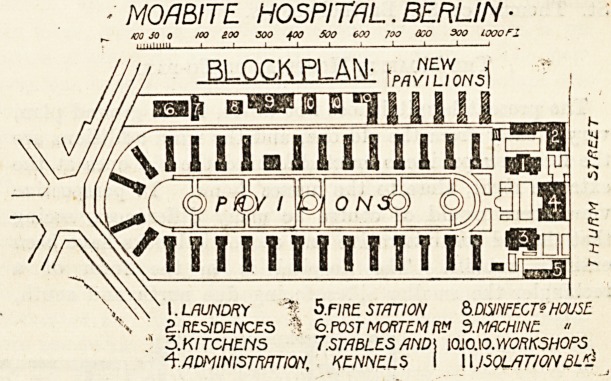


**Figure f2:**
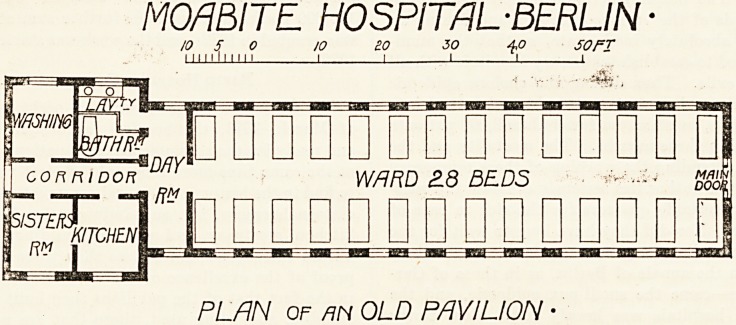


**Figure f3:**